# AbsorbanceQ: An App for Generating Absorbance Images from Brightfield Images

**DOI:** 10.6028/jres.126.039

**Published:** 2022-01-04

**Authors:** Stephen M. Zimmerman, Carl G. Simon, Jr., Greta Babakhanova

**Affiliations:** 1 National Institute of Standards and Technology, Gaithersburg, MD 20899, USA

**Keywords:** absorbance microscopy, image processing, cell viability

**Software DOI:** https://doi.org/10.18434/mds2-2423

**Software Version:** 1.0

## 1 Summary

The AbsorbanceQ app converts brightfield microscope images into absorbance images that can be analyzed and compared across different operators, microscopes, and time. Because absorbance-based measurements are comparable across these parameters, they are useful when the aim is to manufacture biotherapeutics with consistent quality. AbsorbanceQ will be of value to those who want to capture quantitative absorbance images of cells. The AbsorbanceQ app has two modes - a single image processing mode and a batch processing mode for multiple images. Instructions for using the app are given on the ‘App Information’ tab when the app is opened. The input and output images for the app have been defined, and synthetic images were used to validate that the output images are correct. This article provides a description of how to use the app, software specifications, a description of how the app works, instructive advice on how to use the tools and a description of the methods used to generate the software. In addition, links are provided to a website where the app and test images are deployed.

## 2 Introduction

**Fig. 1:**
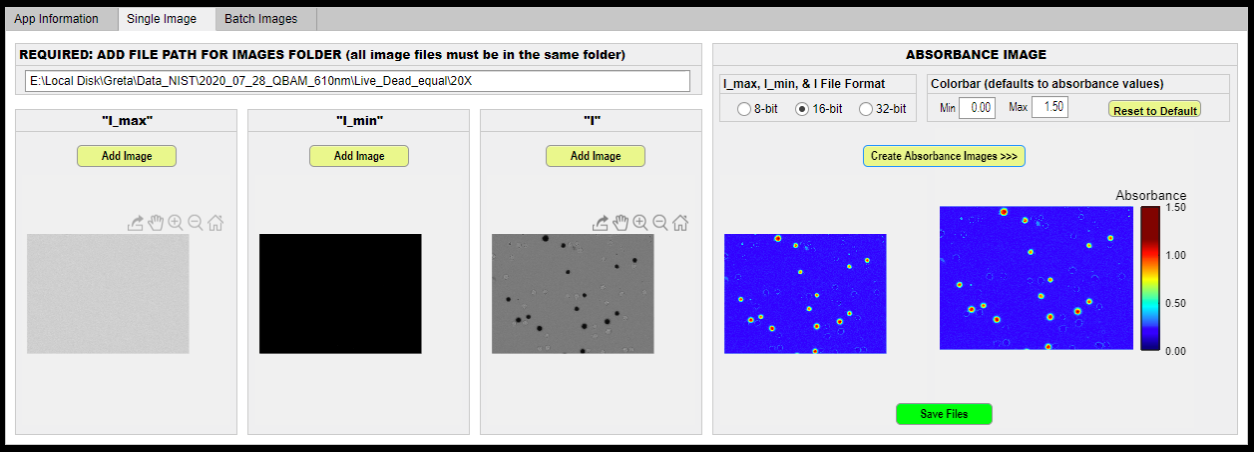
Screenshot of the AbsorbanceQ app illustrating the input brightfield and output absorbance images.

Absorbance microscopy uses a brightfield microscope to capture quantitative images where each image pixel value is in absorbance units [1, 2]. Each sensor in the camera array serves as a tiny spectrophotometer generating a matrix of absorbance values. Absorbance images are more quantitative and comparable than regular brightfield images, whose pixel values represent light intensity values that are difficult to compare due to variability between microscopes, optics, cameras, filters, exposure times, light intensities, and other factors. Quantitative absorbance microscopy is useful for cells that have been stained with light absorbing dyes. An example is trypan blue (TB), which stains dead cells with a ruptured plasma membrane [3]. It is also applicable for imaging cells that express pigments, such as melanin in retinal pigment epithelium [1] or hemoglobin in red blood cells (RBCs) [4]. Herein, the AbsorbanceQ app (general application) (Fig. 1), which transforms brightfield microscope images into absorbance images to enable comparability, is introduced (4).

### 2.1 Absorbance Calculations

Absorbance (A) for each pixel in an image is calculated using Eq. (1)

$A=-{log}_{10}\left(\frac{I\;–\;I_{min}}{I_{max}\;–\;I_{min}}\right),$ (1)

where

i) I is the image capture of the sample,
ii) $I_{min}$ is the image capture with light shutter closed which determines the digital signal in the absence of incident light, and
iii) $I_{max}$ is an image capture of the background, which is the empty stage with the light source on and light shutter open. This image determines the maximum amount of light transmittance. It is equivalent to a “blank” sample that is measured when using a spectrophotometer. The choice of blank is important for getting the best possible results and should be selected based on how the data will be analyzed. Consider the example of trypan blue stained cells on a glass slide. Depending on how the data will be analyzed, the blank could be a glass slide with buffer or a glass slide with buffer containing trypan blue.
- *Note:* The terms I, $I_{max}$, $I_{min}$ are interchangeably used to refer to both the images as well as the pixels within the images. For Eq.1, the terms refer a given pixel of coordinates *x* and *y* within the corresponding images, and the calculations are performed for each pixel position within a set of images I, $I_{max}$, $I_{min}$.

**Fig. 2:**
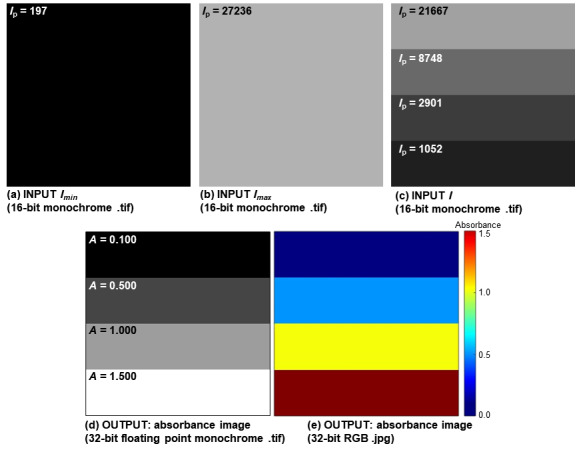
(a-c) Input synthetic $I_{min}$,$\;I_{max}$ and I images with given intensity values ($I_{p}$) used to validate the AbsorbanceQ app. (d-e) AbsorbanceQ output data: (d) 32-bit floating point monochrome absorbance .tif image (pixel values are absorbance units) and (e) an RGB color .jpg image with an absorbance color scalebar.

**Fig. 3:**
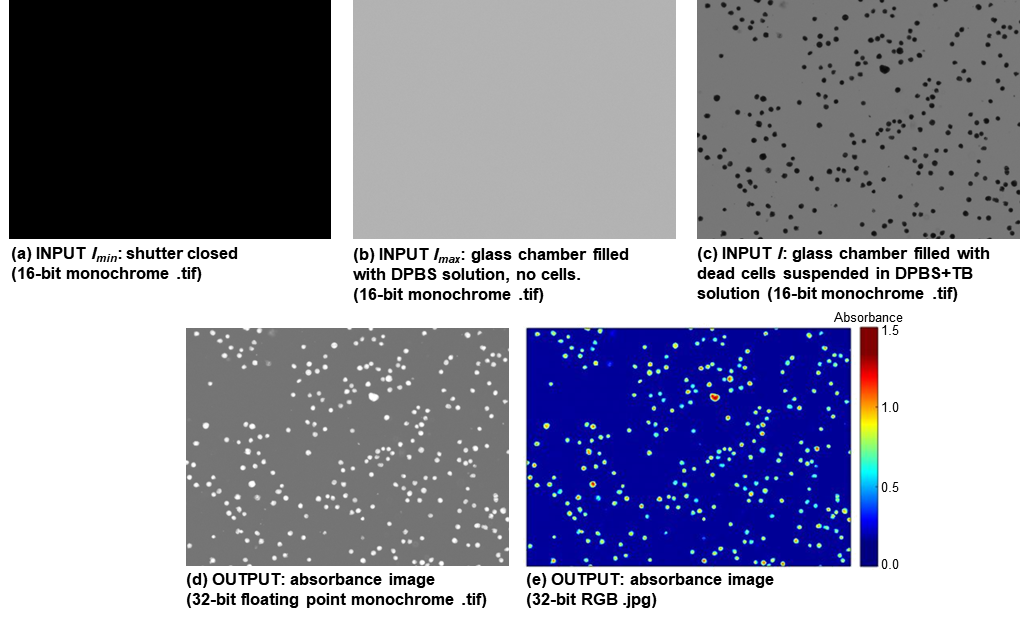
Absorbance imaging of dead Jurkat cells stained with TB. (a-c) Input $I_{min}$ (image with a light shutter closed),$\;I_{max}$ (image of a blank: glass chamber slide filled with DPBS solution) and I (sample image, glass chamber slide filled with dead Jurkat cells suspended in DPBS+TB solution. (d-e) AbsorbanceQ output data: (d) 32-bit floating point monochrome absorbance .tif image (pixel values are absorbance units) and (e) an RGB color .jpg image with an absorbance color scalebar. The dimensions of the images are 793 *µ*m by 586 *µ*m.

These three brightfield images (I, $I_{max}$, $I_{min}$) are required to generate an absorbance image. Corresponding pixels in each of the three input images are used to calculate the absorbance value for corresponding pixel in the output image. For each input image I, AbsorbanceQ outputs two images: 1) a 32-bit floating point monochrome .tif where each pixel represents an absorbance value (Fig. 1, 2d, 3d), and 2) 32-bit RGB color .jpg image containing a color scalebar (Fig. 1, 2e, 3e).

## 3 Software Specifications

**Table:** 

**NIST Operating Unit(s)**	Engineering Laboratory, Energy and Environment Division, Indoor Air Quality and Ventilation Group Material Measurement Laboratory, Biosystems and Biomaterials Division, Biomaterials Group
**Category**	Image processing application
**Targeted Users**	• Researchers, engineers, scientists, cell biologists, students, technicians, tissue engineers, regenerative medicine stakeholders, biomanufacturers • People who measure cell viability via TB dye exclusion or use other cell staining pigment tests • This app will be of value to researchers who want to capture brightfield images that can be analyzed and compared across different operators, microscopes and time
**Operating System(s)**	Windows 10, 64-bit
**Programming Language**	MATLAB (version 9.6.0.1072779, 64-bit Win64, 2019a, MathWorks)
**Inputs**	• There are three input brightfield image files (defined above): I, $I_{max}$, $I_{min}$. The hardware/software settings and shutter speed should be selected so that intensity values are in the linear range of the camera and are not over/under-exposed. I, $I_{max}$ and $I_{min}$ should be taken using the same settings (e.g. exposure time, gain). However, the resultant absorbance image will still be quantitative with different choices of acquisition settings for I, $I_{max}$, $I_{min}$ [1]. • All images must have the same format and pixel dimensions • Acceptable input image formats: i) 8-bit monochrome .tif file (uncompressed) ii) 16-bit monochrome .tif file (uncompressed) iii) 32-bit floating point monochrome .tif file (uncompressed) *Note: .*tif was selected as input since the data is not compressed, which makes it useful for scientific analysis, and because most microscopes can yield .tif files.
**Outputs**	The app yields two output files for each input I image: i) A 32-bit floating point monochrome .tif file whose pixel values are in quantitative absorbance units. This image is suitable for scientific analysis since it is not compressed. ii) A 32-bit RGB color .jpg file with a color scale that is suitable for presentations, Jpg is good for presentations since it is compressed, but it may not be suitable for scientific analysis. *Note:* the output files are saved in the same folder as the input files and are named “Absorbance_Colorbar-filename.jpg” and “Absorbance_Monochrome-32bit-filename.tif”.
**Downloads**	Downloads available here [5]: https://doi.org/10.18434/mds2-2423 The following materials are available for download: • AbsorbanceQ app: downloadable .exe file (1 GB) that will install the app on your computer to occupy 210 MB of disk space • Source code • Synthetic images to verify that the app is functioning properly • Cell images for demonstrating how the app works with experimental data
**Accessibility**	N/A
**Disclaimer**	https://www.nist.gov/director/licensing

## 4 Validation

### 4.1 AbsorbanceQ’s Place in an Analytical Workflow for Absorbance Imaging of Cells

The analytical workflow for absorbance imaging of cells may contain many steps such as experimental design, sample preparation, brightfield imaging, conversion of brightfield images to absorbance images, segmentation of absorbance images, moles of TB inside cells, calculation of cell shape metrics, numerical data analysis, and statistics. The AbsorbanceQ app only performs one of these steps: converting brightfield images to absorbance images. After image capture ($I,\;I_{max},\;I_{min}$) on the user’s microscope, AbsorbanceQ generates absorbance images for each input I brightfield image. Quantitative absorbance images can then be further analyzed in another software (*e.g.*, ImageJ, MATLAB)^1^1 Certain commercial software is identified in this article in order to specify the experimental procedure adequately. Such identification is not intended to imply recommendation or endorsement by NIST, nor is it intended to imply that the materials or equipment identified are necessarily the best available for the purpose..

A key advantage of absorbance microscopy is that data collected on different imaging systems can be analyzed with the same analytical workflow and compared. For image-based measurements, the main bottleneck is usually the establishment of the image analysis workflow. Large amounts of data (*e.g.,* terabytes) can be collected in a few days or weeks using automated imaging systems [1], but the analysis can take years. Further, the analytical workflow used for one microscope may not work for another microscope, since the attributes of the images collected on different systems may be different. An advantage of using absorbance images is that they are intrinsically normalized so that an image analysis process developed for data collected on a given microscope system can be used to analyze absorbance images from other systems.

### 4.2 Validation of AbsorbanceQ App

To validate and test the accuracy of the AbsorbanceQ output, synthetic images were created in MATLAB (version 9.10.0.1602886, 64-bit Win64, R2021a, MathWorks). Synthetic images were created in three formats: 1) 16-bit monochrome .tif, 2) 32-bit floating point monochrome .tif, and 3) 8-bit monochrome .tif. The synthetic images had uniform dimensions (2048 pixels x 2044 pixels, width x height). Pixel values for the images were selected to mimic pixel intensity ($I_{p}$) values observed in data collected for TB-stained dead cells [2]. A range of images were created where each pixel had a preset $I_{p}$ value chosen to yield absorbance values between -0.1 and 1.5. The synthetic images are available for download [5].

Table 1 shows the $I_{p}$ values for the synthetic images (input) and AbsorbanceQ output absorbance values. Since a brightfield cell image (I) can have pixels that are brighter than the corresponding pixels in the blank image $\left(\;I_{max}\right)$, the absorbance calculations (Equation 1) will yield absorbance images with pixels that have negative values. Note that this is not the same as brightfield pixel intensity values being negative. This can happen due to lensing effects (along cell edges), light reflections/refractions on curved surfaces (bubble, dust, or debris), and random intensity fluctuations. This situation was validated for AbsorbanceQ by using a synthetic image that yields negative absorbance A=-0.1 (Table 1). The expected values of A for each image were manually calculated using Eq. 1. The “AbsorbanceQ calculated A” column in Table 1 gives the output A pixel values for the when AbsorbanceQ was used to generate absorbance images from the synthetic images. The difference between the manual calculations and the AbsorbanceQ output was less than 0.001 absorbance units for all cases.

An example of validation with a synthetic image that has four horizontal stripes is shown in Fig. 2, where the synthetic $I_{min}$,$\;I_{max}$ and I images are shown in Fig. 2a-c and the two output absorbance images are presented in Fig. 2d-e.

**Table 1:** AbsorbanceQ app validation using synthetic .tif images.^a^

**File format**	***I*_p_ value of synthetic image *I***	**Manually calculated *A***	**AbsorbanceQ calculated *A***
16-bit monochrome .tif	21667	0.100	0.100
	8748	0.500	0.500
	2901	1.000	1.000
	1052	1.500	1.500
	34237	-0.100	-0.100

32-bit floating point monochrome .tif	0.35420	0.101	0.101
	0.14100	0.506	0.506
	0.04459	1.027	1.027
	0.01410	1.601	1.601

8-bit monochrome .tif	80	0.500	0.500

^a^ The values for the synthetic $I_{min}$ and $I_{max}$ images are as follows: 16-bit $I_{min}$= 197 and $I_{max}=27236$; 32-bit $I_{min}=0.00300$ and $I_{max}=0.44590$; 8-bit $I_{min}=1$ and $I_{max}=251$. *A* = Absorbance. Values in 3^rd^ column were calculated manually and values in 4^th^ column were acquired from AbsorbanceQ output images.

### 4.3 Use Case: Absorbance Microscopy for TB Cell Viability Measurement

An example of using AbsorbanceQ to generate absorbance images from brightfield images [2, 6] is given in Fig. 3. The example is Jurkat cells (Jurkat clone E6-1 cell line; ATCC, Cat.# TIB-152, non-adherent suspension cell cultures) that were killed by a heat-shock treatment (10 min in a 70˚C dry bath) and stained with TB (0.833 mmol/L in Dulbecco’s phosphate buffered saline, DPBS). Trypan blue is excluded from live cells with intact membrane but stains dead cells with leaky membranes. Dead cell suspensions (10 *µ*L) were pipetted into a glass chamber slide (NC-Slide, A8, Chemometec) and imaged with a brightfield microscope. Brightfield images were collected using a 10x objective through a λ = 610 nm bandpass filter (Thorlabs, Cat. # 610-10, full width half maximum = 10 nm). The wavelength of 610 nm was selected because it is near the TB absorbance peak [2]. $I_{min}$ is an image with the microscope shutter closed (Fig. 3a), $I_{max}$ is an image of a blank: glass chamber slide filled with DPBS solution (Fig. 3b), I is the sample image: glass chamber slide filled with Jurkat cells suspended in DPBS+TB solution (Fig. 3c). For each input image I, AbsorbanceQ outputs two absorbance images: absorbance 32-bit floating point monochrome image and an RGB color image with an absorbance color scalebar (Fig. 3d-e). Trypan blue absorbance measurements can be used to determine intracellular TB concentration (moles/cell or molarity) in each cell by employing the Beer-Lambert law [2].

## 5 Applications of Absorbance Imaging

Absorbance microscopy may be helpful for many applications in biomedical science. The example shown in Fig. 3 demonstrates how absorbance images were employed to assess cell viability by measuring the amount of TB taken up by dead Jurkat cells [2, 6]. Retinal pigment epithelium (RPE) expresses melanin as the cells mature and absorbance microscopy can be used to quantitatively and comparably track patterns of melanin expression as a quality metric for tissue engineered RPE implants [1]. Red blood cells (RBCs) express hemoglobin which is a red pigment. Absorbance microscopy may be useful for quantifying hemoglobin in RBCs, especially as induced pluripotent stem cells are used to biomanufacture RBCs. Histology is widely used in research and diagnosis and tissues are often stained with hematoxylin and eosin dyes. Absorbance microscopy could be used to make histological imaging more comparable and quantitative. Users that want to make quantitative microscopy-based measurements can use AbsorbanceQ to transform brightfield images into absorbance images to achieve traceability and comparability in their data.
